# Supplementary material for: Causal Relationship between Aging and Anorexia Nervosa: A White-Matter-Microstructure-Mediated Mendelian Randomization Analysis
Source: Biomedicines. 2024 Aug 16;12(8):1874. doi: 10.3390/biomedicines12081874 (PMC11351342; doi:10.3390/biomedicines12081874)
Supplement: Supplementary file 1 [file biomedicines-12-01874-s001.zip › Supplementary Figure/Figure S4/Singe SNP plot of MR analysis for TL to ubm-b-1974.pdf]

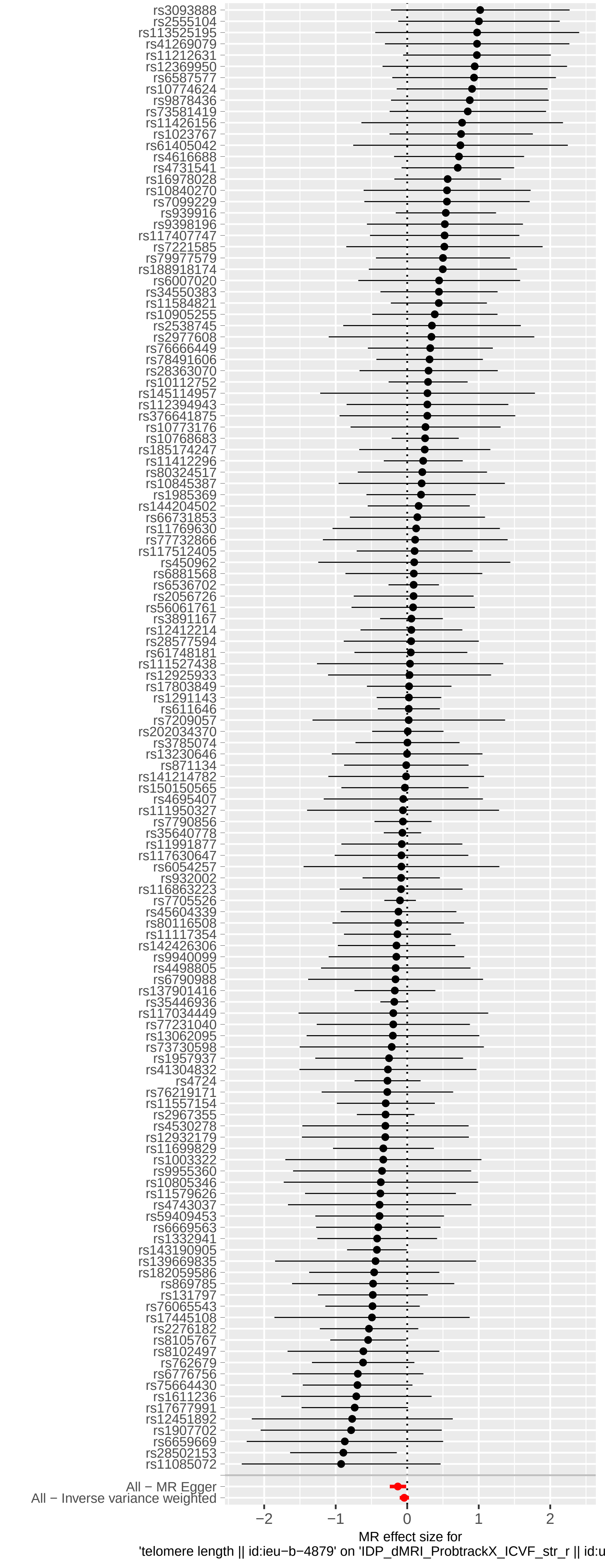
rs3093888
rs2555104
rs113525195
rs41269079
rs11212631
rs12369950
rs6587577
rs10774624
rs9878436
rs73581419
rs11426156
rs1023767
rs61405042
rs4616688
rs4731541
rs16978028
rs10840270
rs7099229
rs939916
rs9398196
rs117407747
rs7221585
rs79977579
rs188918174
rs6007020
rs34550383
rs11584821
rs10905255
rs2538745
rs2977608
rs76666449
rs78491606
rs28363070
rs10112752
rs145114957
rs112394943
rs376641875
rs10773176
rs10768683
rs185174247
rs11412296
rs80324517
rs10845387
rs1985369
rs144204502
rs66731853
rs11769630
rs77732866
rs117512405
rs450962
rs6881568
rs6536702
rs2056726
rs56061761
rs3891167
rs12412214
rs28577594
rs61748181
rs111527438
rs12925933
rs17803849
rs1291143
rs611646
rs7209057
rs202034370
rs3785074
rs13230646
rs871134
rs141214782
rs150150565
rs4695407
rs111950327
rs7790856
rs35640778
rs11991877
rs117630647
rs6054257
rs932002
rs116863223
rs7705526
rs45604339
rs80116508
rs11117354
rs142426306
rs9940099
rs4498805
rs6790988
rs137901416
rs35446936
rs117034449
rs77231040
rs13062095
rs73730598
rs1957937
rs41304832
rs4724
rs76219171
rs11557154
rs2967355
rs4530278
rs12932179
rs11699829
rs1003322
rs9955360
rs10805346
rs11579626
rs4743037
rs59409453
rs6669563
rs1332941
rs143190905
rs139669835
rs182059586
rs869785
rs131797
rs76065543
rs17445108
rs2276182
rs8105767
rs8102497
rs762679
rs6776756
rs75664430
rs1611236
rs17677991
rs12451892
rs1907702
rs6659669
rs28502153
rs11085072
All − MR Egger
All − Inverse variance weighted
−2
−1
0
1
2
MR effect size for
'telomere length || id:ieu−b−4879' on 'IDP_dMRI_ProbtrackX_ICVF_str_r || id:u
